# Make America quiet again: Achieving socially robust knowledge on noise pollution through citizen science

**DOI:** 10.1177/09636625251338190

**Published:** 2025-06-25

**Authors:** Kirsten R. Vegt, Janneke E. Elberse, Bastiaan T. Rutjens, Laurens K. Hessels

**Affiliations:** National Institute for Public Health and the Environment, The Netherlands; Leiden University, The Netherlands; National Institute for Public Health and the Environment, The Netherlands; University of Amsterdam, The Netherlands; Leiden University, The Netherlands; Rathenau Institute, The Netherlands

**Keywords:** citizen science, noise pollution, policy-relevant science, public participation, science attitudes and perceptions, socially robust knowledge

## Abstract

Socially robust knowledge is scientific knowledge accepted by society for its contextual relevance. Citizen science, involving non-professional scientists, offers a promising approach to developing such knowledge. This study examines how citizen science fosters socially robust knowledge through a case-study on noise pollution’s impact on health and well-being in the Dutch village of America. Citizen scientists partnered with researchers of the Dutch National Institute for Public Health and the Environment to study train noise, employing diverse data-collection methods. Interviews with participants revealed trust in this approach and outcomes, contrasting with conventional noise-pollution research. The integration of extended expertise and real-world context, coupled with the project’s iterative feedback loop, ensured that findings were accurate and locally relevant. This case-study underscores citizen science’s potential to create relevant and adaptable policy-relevant science, offering concrete insights into the key elements that contribute to the social robustness of scientific outcomes.

## 1. Introduction

Knowledge helps us understand and change the world around us. Searching for knowledge through science has predominantly been the domain of professional scientists over the past two centuries. However, increasingly, there have been calls for a more integrated approach to knowledge production, involving various stakeholders to tackle complex issues in people’s living environments (Ebel et al., 2018; Gerlak et al., 2023; Gundersen et al., 2022; Irwin, 1995). Within these integrated approaches, a growing need for socially robust knowledge is emphasized, in which social robustness refers to *the societal acceptance of knowledge achieved through its relevance in the context of its application* (Nowotny, 2003; Nowotny et al., 2001; Seijger et al., 2016). It has been argued that a continued reliance on non-socially robust knowledge risks perpetuating an outdated, context-independent view of science, potentially obstructing solutions to real-world challenges and hindering adequate scientific responses to complex societal issues (Allen, 2020; Nowotny, 1999; Weichselgartner and Truffer, 2015).

While definitions of citizen science (CS) can vary, it can broadly be understood as a way to actively involve citizens in scientific endeavours, that generate new knowledge or understanding (European Citizen Science Association (ECSA), 2015; Haklay et al., 2021). It can encompass different levels of engagement and involve various activities in which citizens and scientists work together, such as formulating research questions, creating study plans, and collecting data (Göbel et al., 2021). Technology-driven advancements, including sensor technologies and smartphone apps, enable widespread citizen engagement in data collection and research of the living environment (Berti Suman and van Geenhuizen, 2019; Mazumdar et al., 2018). Ideally, CS balances between generating rich, contextual knowledge while also upholding scientific rigor and credibility (Davies and Mah, 2020).

The promises and claims surrounding CS suggest it may function as a pathway to socially robust knowledge. For example, regarding the “*societal acceptance of knowledge*,” recent studies have indicated that a CS approach can foster trust in science and expert information, by demonstrating transparency and inclusivity in the research process (Bedessem et al., 2021; Hubbell et al., 2018; Thornton and Leahy, 2012; Vegt et al., 2023). While trust is not the sole factor influencing the (societal) acceptance of knowledge and its potential consequences, it is considered an important factor in philosophical discourse surrounding this topic (Quine and Ullian, 1970). Distrust in conventional knowledge regarding the living environment can be driven by both the nature of the knowledge or its “production process” that can be deemed as untrustworthy, as well as psychological drivers and dynamics (Berti Suman and Alblas, 2023; Davies and Mah, 2020; Gundersen et al., 2022; Skarlatidou et al., 2024). Regarding “*relevant knowledge in the context of its application*,” the intention of CS is to include citizens to engage in scientific activities, which enriches research outcomes and increases responsiveness to local concerns (Bonney et al., 2016; Brouwer and Hessels, 2019; Volten et al., 2018), thereby offering knowledge that can potentially lead to effective solutions in people’s living environments (Berti Suman and van Geenhuizen, 2019). While much of the debate on CS is rooted in Western contexts, studies have also explored its relevance in non-Western settings and among indigenous communities, particularly in the context of environmental monitoring and community-based research. Understanding the diverse applications of CS across different geographical and cultural contexts is crucial to assessing its potential for generating socially robust knowledge (Brondízio et al., 2021).

In spite of the many promises of CS, the empirical evidence for its contribution to developing socially robust knowledge and improving societal acceptance is still limited. This article explores these aspects from a social science perspective through a CS case-study in the Netherlands. In the following sections, we theoretically explore the concept of socially robust knowledge in relation to CS. Next, we examine aspects of CS that may be essential for generating socially robust knowledge. We provide context for the case-study, explain the need for socially robust knowledge there, and formulate the research questions that align with the current study’s aims.

### On socially robust knowledge

Nowotny et al. (2001) describe a shift between different modes of knowledge production: from conventional academic research (Mode 1) to a more collaborative and applied approach (Mode 2), with the delivery of socially robust knowledge being a key goal of Mode-2 knowledge production (Gibbons et al., 1994; Nowotny et al., 2001). Mode 2 emphasizes transdisciplinarity, with researchers collaborating across disciplines and engaging non-experts, including citizens, in the process. It prioritizes solving so-called “real-world” problems and acknowledges the contextual nuances of complex issues. Later, Nowotny (2003) proposed three interconnected elements that need to be present in the research approach for knowledge to be socially robust. First, it involves extended expertise by engaging diverse stakeholders, including experts, users, and laypersons, in a transdisciplinary research process. Second, it requires conducting studies in real-world settings, where social, economic, cultural, and political factors add to knowledge development. And third, it should follow an iterative process of testing, modifying, and refining knowledge to ensure its relevance and applicability in specific contexts. This would then, supposedly, lead to socially robust knowledge.

Despite this clarification, the operational definition of socially robust knowledge has faced criticism for its ambiguity on both its conceptual and normative positioning (Hessels and Van Lente, 2008; Polk, 2014; Weingart, 2008). Although the three criteria for socially robust knowledge are generally accepted as plausible, an in-depth understanding of how they precisely lead to socially robust knowledge is lacking. We aim to contribute to this by an empirical exploration in the CS domain. We conceptually build on a study by Seijger et al. (2016), in which the foundational framework proposed by Nowotny (2003) was applied in a case-study, contextualizing the three proposed key aspects essential for achieving socially robust knowledge.

### CS and socially robust knowledge

CS can be initiated bottom-up (by citizens themselves) or top-down (by professional scientists). The degree of participation and influence in a CS process can vary (Haklay, 2018), encompassing activities such as developing research questions and hypotheses, data collection and analysis, drawing conclusions, and disseminating data (Land-Zandstra et al., 2021). Bonney et al. (2009) developed a widely-used framework for categorizing CS-projects. In the most participatory category, co-created projects, citizens are fully involved in all stages of the scientific process, working collaboratively with scientists to design and execute the project. Collaborative projects also engage citizens, but to a lesser extent, allowing them to adjust protocols, draw conclusions, and suggest new research directions. In contributory projects, scientists design the project, and participants mainly focus on data collection and analysis following predefined protocols. CS-projects, especially co-created ones, often align (at least theoretically) with the criteria for socially robust knowledge that Nowotny (2003) formulated: they typically involve citizens as extended expertise, research is usually being conducted in real-world settings in local case-studies, and often an iterative process is followed (Pelacho et al., 2021; Senabre Hidalgo et al., 2021).

Citizens who worry about issues in their living environment are increasingly turning to their own citizen-science measurements (also called “citizen sensing”) to demonstrate that the problems they experience in their living environment are real and serious, even when no (governmental) environmental standards are exceeded (Berti Suman and Alblas, 2023; Kenis, 2020). CS-projects initiated by these concerned citizens often follow a bottom-up approach and/or feature high levels of citizen participation (Allen, 2020). For example, citizens working together with scientists while using affordable, user-friendly, and increasingly high-quality sensors to determine air quality or noise levels in their surroundings (Liu et al., 2021; Volten et al., 2018; Woutersen et al., 2022). The dissatisfaction or distrust of conventionally generated knowledge that leads citizens to measure their living environment themselves, can be directed at the government or offending companies, but also to the environmental standards and the scientific research conducted to model or monitor them (Allen, 2020; Berti Suman and Alblas, 2023; Berti Suman and van Geenhuizen, 2019; Davies and Mah, 2020; Houtkamp et al., 2021; Vegt et al., 2023). From a policy perspective, CS-data is mostly viewed as a scientific tool; it remains largely misaligned with existing policy structures and agendas and is not often used for decision-making on the living environment (Hecker et al., 2019).

### Socially robust knowledge: CS and noise pollution

A key environmental and societal issue where CS has gained relevance and where there might be a need for more socially robust knowledge is noise pollution. Noise pollution is a growing concern worldwide due to its adverse effects on public health and well-being, contributing to issues such as sleep disturbance, cardiovascular disease, and decreased quality of life (World Health Organization (WHO), 2018). In the Netherlands, environmental standards for railroad noise are typically monitored using calculations and models that rely on various parameters, averages, and national monitoring points. However, public acceptance of this approach has decreased, particularly among those affected by noise pollution, extending beyond railways to other sources of noise as well (Welkers et al., 2020). With sound levels primarily being calculated using a virtual model rather than measurements in real-world settings, there is a perceived disconnect between citizens’ own local experiences and the methods employed in addressing the issues they face (Berti Suman and van Geenhuizen, 2019; Shim et al., 2016; Simons et al., 2022; Volten et al., 2018; Welkers et al., 2020). For example, an important critique is that knowledge is mostly being calculated instead of measured and how it focuses on average noise but does not take other factors, such as peak noise levels, sufficiently into account (De Gruijter et al., 2019; Volten et al., 2018). In addition, noise monitoring methods are legally fixed, making it difficult to quickly adapt them based on feedback from those affected by the noise or evolving circumstances (Welkers et al., 2020). All these factors can lead citizens to harbor suspicions that economic interests take precedence over their health and the overall quality of their living environment in policy, a sentiment seemingly reinforced by the methods employed (Houtkamp et al., 2021).

### The case-study of America

**Figure 1.**
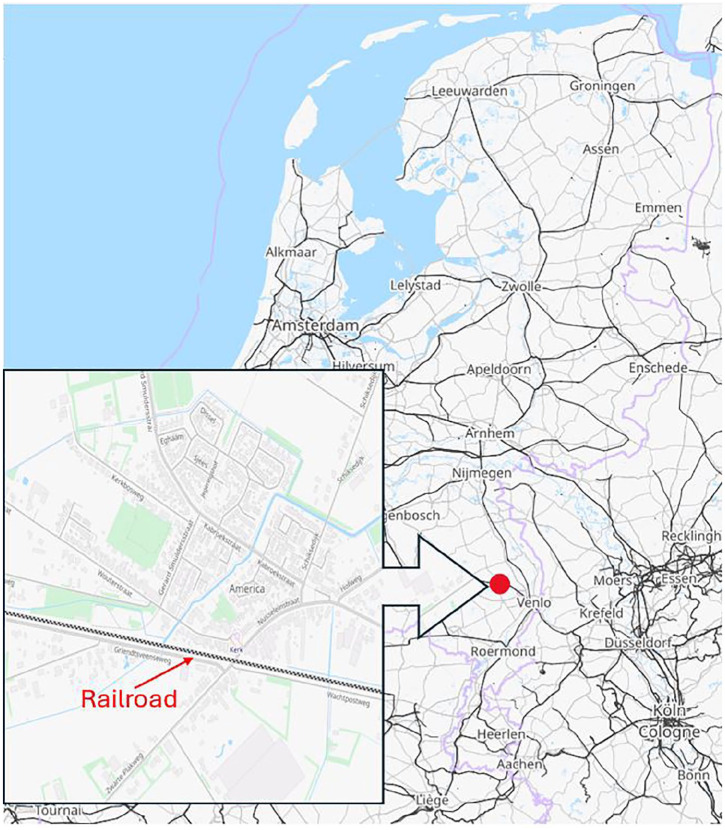
Location of America (Limburg) in the Netherlands. Source: Open Street Map 2024-06-27 3:08 pm (CEST)—data available under ODbL 1.0 (https://www.openstreetmap.org/copyright).

In exploring the concepts of socially robust knowledge and CS, we examine a case-study on railway noise pollution in the village of America (The Netherlands—see Figure 1) where trains pass close to residences. America is a so-called “church village” (*kerkdorp*) in the municipality Horst aan de Maas, which means the buildings (mainly single-family homes and low-rise buildings) are traditionally centered around a single church. The village spans 25 km² and had 2200 residents in 2023 (Centraal Bureau voor de Statistiek, 2024). Over the past decade, residents of America experienced increased noise annoyance due to more frequent, longer and heavier (freight) trains on this route, especially during the night. Citizens feel that it negatively impacts their living environment and well-being.

Residents of America had been frustrated and skeptical about how authorities approached the problem of noise pollution for years, particularly dissatisfied with how knowledge had been gathered and used for policy. Residents believed that actual noise exposure was much higher than indicated by models and felt that their experiences of noise and vibrations from passing trains were not adequately addressed in research and policy. They questioned whether the model-based scientific approach might lead to misguided local noise-pollution policies and sought to generate knowledge on noise pollution and its effects on daily life from (freight) trains that adequately reflected their experiences. Through contact with researchers from the RIVM^1^ for a different project, citizens were connected with RIVM researchers who had expertise in CS and pollution in the living environment. After conversations and discussions (see the Method section for timeline), this resulted into a collaboration between citizens and scientists to research the noise pollution caused by (freight) trains passing by and its effect on people living nearby. The project began mid-2020 and concluded in January 2024.

Through co-creation, a bottom-up CS project was carried out. Residents of America partnered with the RIVM for a CS-initiative to conduct their own research. Together, citizens and scientists defined research topics and questions, developed a noise measurement plan, deployed sound meters, used an app (BelevingApp) to report noise annoyance events, analyzed data, and drew conclusions. The process and outcomes of the project were thoroughly documented, and participants were interviewed to reflect on the scientific knowledge they gathered and how it compared to existing knowledge.

In this project, the RIVM adopted a facilitative role toward the citizen scientists, as the primary goal was to explore and support CS-processes. The emphasis of the project was on learning how to create socially robust knowledge through CS, rather than strictly pursuing technical findings or predefined end points (which emerged nonetheless and were valuable results as well). The scientists could therefore focus on fostering an equitable partnership with citizens and putting their research questions central. The main research question guiding this research is: How can CS, and the way it is conducted, contribute to the development of socially robust knowledge on noise pollution, by (1) expanding the expertise through involving citizens in the project, (2) contextualizing the research in a real-world setting, and (3) fostering an iterative process?

## 2. Method

**Figure 2.**
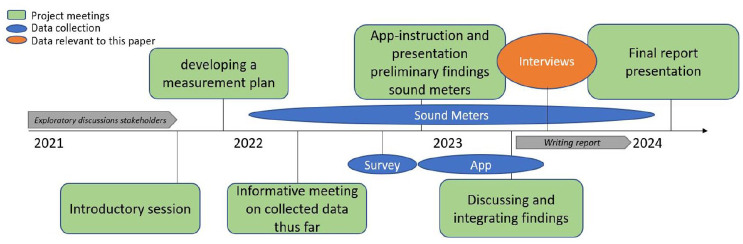
Overview project activities and data collection. Schematic representation of the phases of the project.

The project encompassed numerous activities and moments of data collection. In order to provide an overview of the project’s progression, the following section offers a description of project meetings, key activities, and milestones (see Figure 2).

### Project overview

Mid-2020, exploratory discussions began among stakeholders—including residents, the municipality, RIVM, and the railway organization (responsible for the management, maintenance, and development of the national railway infrastructure)—to address railway noise in America. By January 2022, a citizen scientist network had deployed 18 sound meters across the village, with real-time data accessible via RIVM’s online platform (Samen Meten—Dataportaal). Key activities included the introduction and use of the BelevingApp for reporting noise annoyance, a summer 2022 survey for broader perspective on nuisance and well-being, and in October 2022 discussing preliminary findings. The project concluded in April 2023 with a meeting to integrate data and discuss a final report for Dutch policymakers (Vegt et al., 2024), which was published in January 2024. See Table 1 for an overview.

**Table 1.** Description of participatory project activities.

Date	Project activity
Mid-2020	Exploratory discussions with stakeholders (residents, municipality, railway organization, and RIVM to gain insights and preferences regarding the issue.
September 2021	Commencement session in America for interested parties and stakeholders; RIVM and residents both introduced research goals and sound measurement and annoyance intricacies. Assessment of interest and potential citizen scientists for sound meters. Those who were interested could sign up to participate.
December 2021	Online meeting (due to COVID-19 restrictions) with residents to develop a measurement plan for sound meters and other research questions on noise perception. Collaborative decision-making on research questions and location for sound meters.
January 2022	Establishment of a citizen measurement network of 18 sound meters with residents and RIVM; start of real-time data collection. Formation of an app-group for easy communication between RIVM researchers and citizen scientists.
March 2022	Meeting to discuss and interpret collected sound data; introduction of the BelevingApp to document sound experiences and acute annoyance on specific occasions.
Fall 2022	Survey conducted to understand broader group of residents’ general perceptions of noise annoyance and its impact.
October 2022	Online meeting to demonstrate the BelevingApp and discuss findings so far.
November 2022	Joint measurement week with daily (acute) noise reports in App. After that, noise annoyance reports in App at will.
April 2023	Discussion on sound measurements, survey, and App results; citizen scientist input for further analysis.
April-May 2023	Interviews with participants to gather their perspectives on the project and CS.
November 2023	Feedback of two actively involved citizen scientists on draft research report for policymakers.
January 2024	Publication of research report and public meeting with participants, politicians, and press.

### Recruitment of interview participants

All 18 residents equipped with a sound meter were invited to participate in an interview, and 11 accepted the invitation. Prior to the interview, participants received a written informed consent form, ensuring anonymity and compliance with EU privacy laws. However, in CS projects like this one, balancing privacy with shared agency can be challenging. Naming the village was not an issue for participants, but to ensure accurate representation of the citizen scientists, two of them reviewed the final report in Dutch (Vegt et al., 2024). Participation was voluntary, and withdrawal was possible at any time without consequence. Notably, some citizen scientists actively engaged with the press and policymakers, forgoing anonymity. In addition, five RIVM scientists from the project team were interviewed. The interviews took place in April and May 2023 and lasted between 30 minutes and an hour. All interviews were recorded and transcribed with consent. Participant details and demographics are summarized in Table 2.

**Table 2.** Participant overview.

Participant group	Number	Age range	F/M	Additional details
Residents (citizen scientists)	11	50–79 years	5 F/6 M	Time lived in America: 6–57 years
Project scientists (RIVM)	5	36–55 years	4 F/1 M	2 from social sciences, 2 from natural sciences, 1 from both

### Interview guideline

Participants (citizens and scientists) were individually interviewed to explore their personal engagement and experiences with the project (see Supplemental Material 1). The interviews covered participants’ roles in the case-study, their motivations, and their overall expectations and experiences. Key topics included the perceived benefits or barriers of the CS approach, participants’ perception regarding the scientific knowledge generated, and comparisons to conventional scientific approaches. Moreover, participants were asked to provide insights on how the CS approach contributed to the gathering of knowledge, local applicability and on the acceptance of and trust in science. The interviews were conducted by two researchers: the first author (KV) of this paper and an additional RIVM-researcher who had no involvement in the project. To minimize bias, given KV’s dual role as both project leader as well as researcher for the current study, the additional RIVM-researcher conducted the interviews with the project’s scientific team members (excluding KV, but including co-author JE) and with half of the citizen scientists (especially the ones who had frequent contact with KV in their capacity as project leader).

### Analyzing procedures

Researchers conducted a qualitative thematic analysis following the framework by Braun and Clarke (2006) to systematically identify, analyze, and organize themes within the dataset. The analysis process was supported by MAXQDA software, which facilitated data familiarization, code generation, theme identification, and definition. Each interview was independently coded by one of the two researchers involved in conducting the interviews. To enhance the reliability and reduce potential bias, a third independent RIVM-coder, with expertise in qualitative research but unaffiliated with the project, was engaged in the coding process. This third coder performed a final review of all codes, providing an additional layer of scrutiny. Any discrepancies between the initial coders were discussed, with the third coder serving as the final arbitrator.

## 3. Results

The results of this study have been organized within the framework of socially robust knowledge, focusing on the three interconnected elements as proposed by Nowotny (2003): (1) involvement of an extended group of experts, (2) research conducted in a real-world setting, and (3) an iterative process of knowledge development. Within each element, several subthemes emerged, which are illustrated by illustrative quotes (for more illustrative quotes, see Supplemental Material 2). The interconnectedness of the three elements suggests that potential alternative categorizations of subthemes would have been possible. Therefore, the considerations why certain subthemes were categorized within a particular element will be explained. The illustrative quotes have been anonymized and numbered for both the citizen scientists (C1-11) and professional scientists in the project team (S1-5). When speaking of “participants,” we mean both the scientists and citizen scientists.

### Involvement of an extended group of experts

This element focuses on how involving an extended group of citizen-experts and fostering a co-creative atmosphere, inviting and encouraging citizen scientists to share their expertise, enabled effective knowledge exchange. While the positive impacts of this approach were discussed, some barriers were reported as well. Three subthemes emerged:

Involving affected citizens for issue acknowledgment and societal impact
Knowledge exchange and interaction between (citizen-)scientists
Barriers to extending the expertise in research

#### Involving affected citizens for issue acknowledgment and societal impact

The involvement of citizens in the research brought about a sense of acknowledgment of the issue of noise annoyance for participants, where there was not at first:

Of course, it makes a big difference for our community, that we feel we can have a say, that our complaints are taken seriously.—C02

During project meetings, participants were invited to share their worries, concerns, and questions about noise annoyance with the researchers. By creating time and opportunities for citizen scientists to voice their issues, and by giving them decision-making power in designing the project, specific research questions important to the residents were included—questions that would likely have been overlooked if left solely to the scientists.^2^ This approach made the residents feel heard and acknowledged, fostering a sense of ownership of the project:

This is something we’ve done together. We’ve discussed everything together, like ‘what do you want, what do you need from us?’(. . .) That resulted in something we are a part of as well, something that we contributed to. That feeling of doing this together and being acknowledged, that makes so much difference compared to. . . like people who just sit on their throne . . .—C05

The scientists remarked on this as well, noticing the reaction of the participants on the final results of the project. It resonated with the community’s experiences, fostering a sense of validation and acknowledgment of the noise-pollution issue.

Because people recognized their experiences in the final results, this research also feels as a form of acknowledgment of their problems. And that is very important. Because up till now, all they’ve heard is ‘our methods say you don’t have a problem, so stop complaining.’ (. . .) And they get angry because they are not getting their problems acknowledged.—S02

The involvement of affected citizens also influenced the way the case-study connected with society in general. Citizens described how noise pollution affected them or their loved ones in their daily lives. The personal stories of residents were a sort of “reality check” for researchers on the negative way noise pollution affects society, and how it causes real harm. Because of this, researchers felt the case-study became less abstract and more connected and relevant to society:

I think that on the one hand, by doing science or research together with citizens, you are forced to look at many issues much more practically and to think about the local impact it can have. (. . .) And what benefit the research has to people in certain communities. But at the same time, it’s making science or research much more relevant to citizens because it clearly addresses their stories, their issues. So I think it works both ways in that sense.—S05

#### Knowledge exchange and interaction between (citizen-)scientists

Participants noted the CS approach had a positive effect on their interactions as residents, fostering a sense of community and mutual support. All citizen scientists expressed appreciation for the opportunity to collaborate as equals in the project, rather than just being told what to do by scientists. They reported feeling like the exchange of ideas and information could take place in a transparent way, from an equal position or partnership.

We could ask questions whenever we wanted and the explanations were clear. So, what more could we want? We even exchanged datasets.—C08

This was experienced by the scientists as well; instead of being the sole explaining party, they were having things explained to them by the citizens. Therefore both scientists and citizens could participate from a position of strength and knowledge. For the scientists, this mostly meant knowledge about the content of the issue, such as how to conduct sound measurements and measure noise annoyance and perception, and ensure the science being done was sound and robust. For the participating residents, this mostly meant knowledge about the local context, nuances, and their experiences regarding the issue of the noise. So, albeit their positions were different, they were equal nonetheless.

I find it very interesting citizens also explained things to the scientists. (. . .) Ultimately, I think it boils down to a more balanced collaboration, a more equitable collaboration that harnesses the strengths of citizens and professional scientists.—S03

However, despite this feeling of equality, a disparity in self-perception did exist between citizens and professional scientists as well; some citizens reported feeling inadequate compared to scientists. Many citizen scientists were hesitant to call themselves as such because they felt like science was something more imposing than what they were doing in the project.

When I see what my family member does as a scientist, no. No, I think citizen scientist is too grand a term for us.—C03

#### Barriers to extending the expertise

Barriers were reported as well. Professional scientists made remarks about facing skepticism from peers regarding the value of citizen expertise. The amount of time and effort that comes with conducting a CS project like this was mentioned as well.

It also takes much time and effort. And initially, you don’t get funding for that. That is, of course, another problem scientists face (. . .) you are overburdened. You have to do many things, you have to do them all properly, and you want to do them well.—S02

Furthermore, some comments regarding inclusivity were made. Scientists’ remarked the group of citizens that were most involved were pensioned white men, leading to worries whether this project had been sufficiently inclusive of a broader, more diverse range of participants. However, citizen scientists in this case-study who were not part of this demographic did not voice these concerns themselves. Instead, they conveyed a positive attitude toward their more actively involved neighbors, indicating that they felt adequately represented by them.

I felt very involved. (. . .) But I don’t have much time available. I’ve noticed other people who are less busy, who take on a much more active role in the project. That’s a good thing.—C06

In addition, the challenge of balancing scientific rigor with community involvement was felt by some scientists. Relinquishing control to citizen scientists was experienced as daunting by some, since a responsibility was felt of ensuring data validity and preventing biases.

It’s also quite challenging for scientists because you have to relinquish some control [to the citizens]. This control, as I mentioned earlier, is sometimes necessary to ensure that biases and other issues don’t arise. I didn’t expect this initially, but it’s something I’ve noticed throughout the process.—S04

At times, some citizen scientists shared opinions on specialized technical topics that required corrections from scientists. While this did not create a negative atmosphere—project meetings remained constructive and positive—it underscored the need for ongoing scientific involvement to maintain research rigor and validity. Because of this constructive atmosphere, there was mutual respect for each other’s expertise. As a result, when scientists advised against a particular approach and explained why it would be problematic, the discussion quickly evolved into a collaborative dialogue aimed at finding the best solution that satisfied all perspectives. In other words, the intensive co-creation process enabled the quick identification and correction of errors, fostering a valuable learning environment for all involved.

Because citizen scientists can be very smart and sharp about local effects, and they are increasingly involved in recognizing those in data analysis. But we also see that sometimes they make mistakes, which is very understandable if you’re not trained for it.—S01

### Research in a real-world setting

In this section, we explore how doing CS in a real-world setting had an effect on the actual knowledge that was generated, and how participants perceive this “new” knowledge compared to the “old” knowledge. This will be discussed in three subthemes:

Measurements by a CS network vs establishing sound levels by modeling
Sound-level measurements reflecting the real-world setting
Combining sound-level measurements and perception data

#### Measurements by a CS network vs establishing sound levels by modeling

In the context of local noise assessments, participants expressed skepticism toward standardized modeling approaches that did not align with their lived experiences. Some citizen scientists suspected that these models were deliberately used by institutional actors in a malevolent way, to maintain the status quo and allowing them to continue profiting or avoid addressing the problem.

Those calculation models can do whatever, but the sensors don’t lie—C04

However, most participants had a more nuanced view. They believed that the models were simply unsuitable for local use or lacked the necessary detail to accurately represent the problem. Instead, participants valued tangible and detailed noise data that directly corresponded with their experiences, finding such data more trustworthy. This trust was reinforced by the sound meters used in this CS project, which provided the detailed information they sought.

Everyone has their own microphone that produces data, and you can be sure that data is accurate. (. . .) I can check it through an app. I can just check what. . . Especially last night, around four o’clock, I was awake and a huge train went by. I can check on the app, if I want to, how loud that train was—C10

#### Sound-level measurements reflecting the real-world setting

The case-study with sound meters fulfilled the residents’ need for a different, local type of knowledge that had not been gathered yet. For example, residents were curious about specific aspects of the train noise. Using the sound meters to address their research questions satisfied this need in four different ways:

1. Registering peak sound levels validated residents’ personal experiences with the train noise as well as the amount and time of trains passing through the village, providing concrete data to back up their experiences.

You can see those peaks regularly, which makes clear how many trains actually pass by.—C01

2. Distinguishing train noise from other environmental sounds made it possible for the project to focus specifically on train noise, ensuring that the data collected were relevant and precise to residents’ concerns.

Indeed with those filters. . . the other traffic is removed. That was indeed something I had hoped would happen. It’s nice this has been achieved.—C02

3. By capturing noise levels at multiple locations, residents could better understand the spatial variability of the train noise and its impact on different parts of their living environment.

I think that our own measurements are very important and I trust those, because the sound meter is hanging both at the garage behind the house, and inside the house as well.—C06

4. Understanding how noise levels fluctuate under various conditions provided insights into potential noise mitigation strategies, such as reducing train speeds.

Look, if there’s a period when it’s busy on the track or trains are moving slower, then there’s much less noise. Yesterday it was busy on the track and then an iron ore train passed by, a long one, but it was driving slowly. We were all sitting outside, and we said to ourselves, “if they’d just go a little slower, that would improve things a lot.”—C07

From a scientist point of view, it was reported the citizen involvement ensured that anomalies in the data could be promptly identified and contextualized, enabling more accurate interpretations, adjustments, and analysis.

Because those sensors are at their homes all the time, it’s actually a kind of manned measurement. So basically, when strange things happen, citizens know that the measurement isn’t correct and they can report that, for example because there’s another sound source, and then we can take that into account. Or when, at a certain moment no trains unexpectedly ran because of track work, residents immediately noticed that and told us like: hey, there were no trains last night, you can use that as a baseline measurement to see how the situation is when there are no trains running. We couldn’t have seen that as quickly ourselves. So in many ways, citizens are an important part of this.—S03

#### Combining sound-level measurements and perception data

Participants emphasized the importance of capturing individual experiences alongside sound-level data. They felt the integration of citizen perceptions of noise with measurements of sound levels revealed a more comprehensive understanding of the noise annoyance, helping to contextualize the data within the real-world experiences of those affected. It allowed for a more nuanced understanding of the impact of noise, acknowledging that the human experience of noise cannot be fully captured by just measuring sound levels alone.

If the residents participate, you’ll get their experiences, and in this case measurements as well. I think the experiences and the perceptions of people were just as striking and significant. The study on how people experience and perceive the noise provided much insight.—C09

The scientist’s perspective marked a shift in research methodology, moving from broad generalizations to a detailed exploration of individual and community experiences with noise. Rather than focusing on mean noise levels, the study examined how noise affected daily life. Individual experiences were discussed collectively. This collective analysis highlighted the diversity of experiences and revealed broader patterns in the community. The transition from individual to collective (local) understanding offered a comprehensive view that extended beyond isolated experiences.

What is new in particular is that we look at the individual experiences of citizens. (. . .) There are studies that ask very large groups of people about their experiences over the whole year of a certain noise source. So that is much more generalized, and they link those to noise models that are also more generalized. But here we really focus on the individual situation. We place a sound meter at a person’s place and ask how he or she experienced it at a particular time of day. (. . .) We really put the magnifying glass on something else, so to speak.—S03

### Iterative process

For this subtheme of socially robust knowledge, we explored the iterative process involved in producing knowledge through CS, emphasizing how society actively participates in the production and refinement of knowledge. Two key subthemes have emerged:

Short feedback loops and mutual learning
Iterative knowledge integration and innovation

#### Short feedback loops and mutual learning

This subtheme highlights the importance of continuous feedback and interaction among participants throughout the research process. The short feedback loops in the project were instrumental in identifying the core issues that needed to be studied from the residents’ perspective, as well as determining whether and how those issues could be addressed in a scientifically sound manner.

And we have also discussed this with the residents: what the measurements tell us but also what they don’t tell us. It tells us something about the sound, but it doesn’t tell us how it is experienced. So, we need more than that (. . .) They have shown us that something more is needed—S05

Ongoing conversations and discussions between scientists and citizens played a crucial role in mutual learning. Participants gained insight into scientific concepts related to sound measurement, noise annoyance, and research methodology in general. They applied this scientific knowledge to their local context and shared these insights with scientists, who in turn incorporated it into the research project.

[Because of collaborating in the project] we’ve gotten smarter, and you’ve gotten smarter too.—C07

For instance, when discussing sound measurements, participants wanted to include personal noise experiences. In response, citizens and scientists collaborated to integrate these experiences while maintaining scientific rigor, leading to the inclusion of the BelevingApp. Similarly, a broader survey study was introduced in the project to assess village-wide noise annoyance. Although not fully aligned with CS principles—here, citizens were respondents rather than co-researchers—it stemmed from their own research question: “How many and to what extent do people in our village experience this problem?” The results were then shared, discussed, and contextualized with citizens, reinforcing collaboration. Community feedback also improved accessibility of data on the online data portal, enhancing transparency and usability.

The way of making the data accessible has also received feedback and has been discussed in consultation with those who want to use that dataportal. (. . .) So yes, you immediately have a short feedback loop, so to speak.—S04

#### Iterative knowledge integration and innovation

Participants expressed a desire to both expand upon existing scientific knowledge and integrate it with new insights gained from their CS-efforts. They hoped that the knowledge generated through their involvement would serve as “new” knowledge that could refine and inform the scientific community’s understanding of environmental noise, thereby contributing to the advancement of the field. Specifically, they aimed to enhance existing noise models by incorporating citizen-generated data, bridging the gap between established methodologies and real-world experiences. In other words, besides the iterative process that was followed within the CS project itself, participants were also aware of their potential role in the broader scientific process and conversation on this topic. They hoped to contribute to the feedback loop within science, actively engaging in the discussion on what constitutes valuable knowledge about noise pollution within the scientific community.

I hope that those who create those calculation models take this kind of research into account in the update of the calculation model. (. . .) Because it would get closer and closer to reality.—C05

Okay, so we have a sound model, we have sound calculations, we have sound measurements, and we have citizen [sound and perception] measurements. And how do these relate to each other? What can you do with that? If we can start having that discussion, then I think we will make progress, as a science—S01

Furthermore, citizens’ involvement in designing and improving noise measurement instruments showcased the potential impact of CS on technological innovation. In this project, it was demonstrated how citizen-driven initiatives could enhance the accuracy and utility of said measurement instruments.

What many people are interested in is: how well do these CS sound meters measure? (. . .) They are not far off from official class 1 sound meters and are very useful for mapping various things. They are really quite accurate, and cheap, and because of that, you can deploy a lot of them, so you get a better picture of the situation. (. . .) If we would’ve placed two class 1 sound meters in America, we would have had much less useful information about the area. (. . .) And these sound meters designs are also freely, publicly available, designed by citizens. So the design of these sound meters is CS as well. They are also continually improved by citizens, so they keep getting better—S03

However, participants were also aware of the difficulties of reconciling established models with CS-data, highlighting the complexities of integrating “old” and “new” knowledge; for example, the complexities of connecting different types of data and approaches but the impracticality of a more widespread application of the current methodology due to its (current) high costs.

The connection between the measurements we’ve done now and a model seems impossible.—C02

Citizens say, “Why do you use models and why aren’t measurements just taken everywhere?” There are reasons for that, of course (. . .) Sometimes things just can’t be done differently because it would be too expensive, for example.—S04

### CS knowledge acceptance

The results described earlier align with the three conditions outlined by Nowotny (2003). However, participants mentioned other important aspects for the ‘societal acceptance of knowledge in the context of its application’: it must be seen as useful by those in a position to implement change. This underscores that, in this case-study, the relevance of CS knowledge is inherently tied to its ability to influence real-world decisions. If policymakers disregard citizen-generated knowledge, its applicability is diminished, challenging its status as socially robust knowledge. Two key subthemes emerged:

Utilization and advocacy
Tensions and skepticism regarding policy impact

#### Utilization and advocacy

Participants expressed a strong desire for their findings to inform policy and drive noise mitigation efforts. They believed their data strengthened their case for environmental improvements:

And what’s valuable is, you can confront an alderman with it and say: look here. We have taken measurements. (. . .) these are the facts, these decibels are really high here, it’s written in black and white.—C04

In addition, the involvement of scientists in data collection and analysis further reinforced confidence in the findings, particularly regarding their credibility in policy discussions. The association with the RIVM was seen as a mark of reliability, increasing the perceived legitimacy of the results that would enhance the acceptance of their data by policymakers:

I think it only strengthens the outcomes, right? Especially when policymakers say: ’Hey, this has been verified by the RIVM so it must be reliable.’—C11

#### Tensions surrounding policy impact

Despite these advantages, participants were skeptical about whether policymakers would acknowledge their findings. Unlike government-commissioned noise-pollution studies, this CS project operated independently, raising concerns about institutional resistance to alternative data sources and the potential for inaction.

[local] Policymakers have said: we are not going to consider them, we will stick to the calculations. So that is a bit of a shame.—C09

That is where I think people get frustrated with CS. They think, in this case too, I’m going to measure, I’ll show how bad it is, and then something has to change. But nothing happens. Which is super demotivating of course, and next time they’ll think: why bother, participating in science is useless to us. So, a backlash can occur.—S01

At this stage, some tension arose between the RIVM and the citizen scientists. Citizens expected and hoped the report would be formally presented to the national policymakers at the ministry, potentially even the Minister. However, since this was no government-commissioned research, the ministry was informed about the report, but no formal presentation took place and the report was not actively pushed onto government agendas. The report was instead made publicly available on the RIVM’s website.

You said, ‘There will be a report, you [the public] will receive it, and you can do with it what you want.(. . .) It’s not commissioned by the ministry, so they won’t receive it from us necessarily.’ Our thought was: you are here for society, for the Netherlands. And if you discover something, then it’s your responsibility to pass it on—C01

However, after some discussion, there was more understanding regarding the RIVM’s position. After clarifying that citizen scientists were free to make use of their own means to bring the report under the attention of the people they thought needed to see it, they took initiative by organizing an official event to present the findings. This was fully organized by the citizen scientists, for which they invited and included the RIVM, local policymakers, the alderman, municipal council members, and the press—all of them attending (or representatives of them). The RIVM gave a presentation of results, and together with representatives of the citizen scientists, they handed the final report to the alderman.

To summarize, collaboration between citizens and scientists enhanced the credibility and relevance of the project’s findings. While institutional channels for direct policy agenda-setting were limited, the partnership gave citizens greater confidence in the data. Empowered by this validation, they took the initiative to organize an event to spotlight the project and maximize its policy impact.

## 4. Discussion

This study builds on the theoretical foundations laid by Nowotny (2003), further clarified by Seijger et al. (2016), contributing to empirical evidence. Our case-study in the village of America elucidates CS’s role in producing socially robust knowledge. Consistent with the conditions outlined by Nowotny (2003), this study shows how three key aspects of the research in the case-study contribute to the production of socially robust knowledge: (1) expanding the group of experts involved, (2) contextualizing research in real-world settings, and (3) following an iterative process. A fourth aspect of achieving socially robust knowledge through CS emerged as well: the importance of its acceptance by policymakers and its perceived usefulness in driving real-world improvements, underscoring that for knowledge to be seen as relevant in its context of application, it needs to be utilized by those who can implement change.

In line with previous research, involving citizen scientists in the noise-pollution study led to the acknowledgment of noise issues and fostered a sense of ownership and validation among participants (Berti Suman and van Geenhuizen, 2019). Residents’ concerns and local knowledge were expressed through equitable knowledge exchange which shaped the research questions. The real-world setting of the research provided measurement-data that addressed residents’ concerns and questions, validating their own experiences (Berti Suman and van Geenhuizen, 2019). Combining sound meter data with perceptions provided a comprehensive understanding of (short-term) noise annoyance, and contextualized real-world data, making the research locally relevant (Liu et al., 2021; Vohland et al., 2021). As highlighted by Senabre Hidalgo et al. (2021), the short feedback loops and ongoing dialogue in an iterative process, with citizens and scientists leveraging their respective strengths in scientific rigor and community knowledge, facilitated mutual learning and research refinement. However, the uncertain integration of this knowledge into policy affects its relevance. Given the Netherlands’ busy railway network and the EU’s plan to double rail freight by 2050 (European Railway Agency, 2021) and the growing impact of environmental noise on public health (WHO, 2018; Welkers et al., 2020), policies designed to mitigate these negative effects could benefit from incorporating CS, making them better informed and locally adapted (Berti Suman and van Geenhuizen, 2019). Thus, a CS approach would likely enhance policy effectiveness and strengthen the social robustness of the knowledge by ensuring its enduring relevance to the communities affected. In CS-projects where (one of) the aim(s) is to inform public policies, policy acceptance could be considered a crucial step in the path toward making CS truly socially robust. Citizen-led advocacy can engage policymakers, with scientists supporting this process by empowering citizens to do this and facilitate the integration of their findings into policy discussions. While scientists may be cautious about appearing non-neutral in these processes, standing by the validity and reliability of the CS-data is not necessarily advocacy—it is simply upholding good science.

### Barriers and limitations

A key limitation of this study is that the policy follow-up resulting from the CS project remains open-ended; we could not include all the relevant events that took place after the project ended. While participants worked hard to highlight the project’s findings, it is unclear how their views will evolve as the actual impact on policy becomes clear in the future. In addition, as with many qualitative studies, limitations such as a small sample size and the potential influence of researchers on participant responses may have impacted the results (Anderson, 2010).

This CS project benefited from adequate funding and institutional support, allowing the CS process to take center stage without the typical constraints of time, funding, and focus on conventional research methods. This environment fostered collaboration and minimized conflict, as many of the participants’ desires for the CS project were achievable. However, we realize that not all CS research projects have these beneficiary circumstances, and therefore, the findings may not be (completely) generalizable to other settings.

### Reflections and future research

Including the perspectives of residents affected by noise pollution provided critical insights into why citizens sometimes reject conventional scientific approaches and seek alternative forms of knowledge, such as CS. As Nowotny (2003) highlights, questions like “*Who is hurt?*” and “*Who benefits?*” reveal the normative dimensions of scientific research, underscoring the need for diverse perspectives and socially robust knowledge. This is particularly relevant for policy-relevant science concerning people’s living environments, as it is inherently value-laden. Expert framing can (often unintentionally) overlook certain concerns or focus on specific questions while excluding others (Gundersen et al., 2022; Kenis, 2020). Decisions about measurement locations, selected parameters, or timeframes shape scientific observations and their public presentation. While all methodological choices involve trade-offs—broad perspectives may miss local nuances, while narrow perspectives risk fragmentation (Kenis, 2020)—it is crucial to make these decisions transparent rather than presenting conventional methodologies as the only neutral and objective options. Our findings support this perspective: an integrative CS approach can bring environmental issues to light from multiple angles, and help bridge the gap between policy-relevant research and community concerns. Of course, this does not mean that all science is inherently political in the same sense, or that citizen knowledge is inherently *better* than conventional science. However, strict adherence to conventional methods can result in the dismissal of solid CS-data that could actually be helpful to learn more about an issue, which, in turn, may make citizens feel that their concerns are ignored. This sense of exclusion can erode trust in both policymakers and (policy-relevant) science (Skarlatidou et al., 2024). Citizens may feel that if the science that feeds policy does not address their concerns, it serves little purpose for those it is meant to benefit. Therefore, we concur with colleagues in advocating for a broader approach to knowledge production (Ebel et al., 2018; Gerlak et al., 2023; Gundersen et al., 2022; Irwin, 1995), especially when tackling complex environmental issues like noise pollution, *particularly* when they inform policy. Since policymakers often struggle to integrate CS into decision-making (Hecker et al., 2019), future research could focus on effective strategies to help policymakers meaningfully incorporate CS contributions into decision-making processes.

Interestingly, a disconnect between the CS experience and the broader concept of science was also evident in the project. Many citizen participants were strongly reluctant to identify as “citizen scientists,” viewing the label as an aspiration that felt too lofty. Despite their active involvement in all stages of the research, many did not perceive their contributions as “scientific.” This reflects a broader issue: science as a concept is often seen as distant, specialized, and unattainable, or is not recognized as something that is connected with much of the technologies and items we use in our everyday life (Rutjens and Hornsey, 2024). This perceived distance contributes to science skepticism in various domains (Većkalov et al., 2024) and should be considered within a broader societal context, especially in an era of rising distrust and “alternative facts.” Populist movements worldwide reject or deny evidence of anthropogenic (local) climate change effects, or downplay the harmful effects of pollution in the environment (Davies and Mah, 2020). In contrast, CS—when based on rigorous methodology, transparency, and collaboration—can be a promising approach to counter misinformation and foster evidence-based discussions between stakeholders on environmental issues (Allen, 2020; Vegt et al., 2023). Therefore, future research could further explore perceptions of (perceived distance to) science and scientific practices within CS contexts. Beyond examining the knowledge production process, studying individuals who take on the role of citizen scientists and the psychological factors influencing their trust in and acceptance of scientific knowledge could provide valuable interdisciplinary insights into public trust in policy-relevant environmental science.

## Supplemental Material

sj-pdf-1-pus-10.1177_09636625251338190 – Supplemental material for Make America quiet again: Achieving socially robust knowledge on noise pollution through citizen science Supplemental material, sj-pdf-1-pus-10.1177_09636625251338190 for Make America quiet again: Achieving socially robust knowledge on noise pollution through citizen science by Kirsten R. Vegt, Janneke E. Elberse, Bastiaan T. Rutjens and Laurens K. Hessels in Public Understanding of Science

## Notes

1 The RIVM (Dutch National Institute for Public Health and the Environment) conducts independent scientific research to promote public health and a sustainable, safe environment and provides expert advice to the government, professionals, and the public.

2 In the paragraph “Research in a real-world setting,” it is elaborated on to which new knowledge specifically these citizen-research questions led.
