# Supplementary material for: Make America quiet again: Achieving socially robust knowledge on noise pollution through citizen science
Source: Public Underst Sci. 2025 Jun 25;34(8):1066–87. doi: 10.1177/09636625251338190 (PMC12535618; doi:10.1177/09636625251338190)
Supplement: sj-pdf-1-pus-10.1177_09636625251338190 – Supplemental material for Make America quiet again: Achieving socially robust knowledge on noise pollution through citizen science [file sj-pdf-1-pus-10.1177_09636625251338190.pdf]

Make America quiet again – Achieving socially robust knowledge on noise pollution through citizen science

Kirsten R. Vegt
National Institute for Public Health and the Environment, The Netherlands
Leiden University, The Netherlands

Janneke E. Elberse
National Institute for Public Health and the Environment, The Netherlands

Bastiaan T. Rutjens
University of Amsterdam, The Netherlands

Laurens K. Hessels
Leiden University, The Netherlands
Rathenau Institute, The Netherlands

SUPPLEMENTAL INFORMATION CONTENTS

1: Interview Guideline [translated] p.2

2: Tables with Illustrative Quotes [translated] p.5

1 – Interview Guideline [translated]

This interview guideline has been condensed for clarity and conciseness. The primary questions and main topics covered during the interviews are summarized to present an overview of the qualitative research process in the interviews.

<u>Citizen scientists</u>

General experiences with the 'Citizen Science Network Rail America' project

You have participated in this project over the past 1.5 years. You have been involved in one or more of the following activities: conducting measurements with sound meters, participating in the measurement week, or attending joint sessions about the measurements.

- How were you involved in this project?
    - To what extent did you feel involved? What contributed to this feeling?
    - To what extent did this project add something of value to you
    - How did this project add to knowledge around the problem of noise annoyance?

'Citizen Science Network Rail America' = Science?

We are curious about your vision on research and science in general.

- How do you perceive science/what comes to mind first when you think of science?
- To what extent do you consider what has been done in this project as science? Why or why not?
- To what extent do you consider yourself a citizen scientist, after participating in this project?
- Based on your experiences within this project, how do you think the collaboration between citizens and scientists makes scientific research different from when scientists work alone?

Trust in (citizen) science based on the 'Citizen Science Network Rail America' project

The following questions concern what has been done in this project (citizen science) to investigate train-related annoyance, compared to the current approach (scientists using models) of conducting such research.

- To what extent do you trust the research on train noise and noise nuisance as it was done previously (using models without citizens) and as it was done in the project (by measuring together with citizens)?
    - The 'who' (RESEARCH INSTITUTE / individual researchers, etc.)
    - The 'what' (Suitability/usability of results, etc.)
    - The 'how' (process/method/tool, etc.)
- Trust in (local) government based on 'citizen science' (à 10 min) To what extent has the project influenced your trust in the (local) government?
    - How the (local) government takes your interests into account.
    - Trust in fair and just assessment of the annoyance.

    - Trust in decision-making/actions concerning annoyance reduction.

Demographic data + conclusion

(with room for the interviewee to add to what they already said on the subject)

<u>Scientists on the project team</u>

Introduction in general

- Can you briefly introduce yourself (academic background, current position, etc.)?
- How (and since when) did you become involved in citizen science projects (focused on environmental monitoring in the living environment)?
- What was your initial impression of citizen science when you first became familiar with it?
- Have things changed in your perception of science due to your participation in citizen science projects?

Experiences with the 'Citizen Science Network Rail America' project

You have been involved in this project for the past 1.5 years. Now follows a number of questions about your general experiences with this project:

- In what way were you involved in this project + role?
- To what extent has the participation of citizens in this research changed the research or 'the science'?
- To what extent do you believe that this research has or will have an impact on policy & what role does citizen involvement play in this?

Impacts of citizen science on science and society

A number of promises/effects are attributed to citizen science in terms of impact on science and society (adapting elements from Brouwer & Hessels, 2019).

- Could you reflect from your own experiences with CS in the project on the following statements:
    - Citizens can help collect or analyze more data, thereby increasing the quality of science in general.
    - Citizen involvement increases the relevance of research agendas for the broader society.
    - Citizen participation can help 'correct' skewed representation in knowledge production (by including underrepresented groups)
    - When citizens are involved in research, the perspectives included are more diverse than in research where this does not happen.
    - CS can enrich the quality control mechanisms of science through 'extended peer review'
    - CS increases scientific literacy among citizens, allowing them to gain knowledge and learn about how science 'works' by conducting their own research.
    - CS can lead to greater public acceptance/trust in scientific knowledge

- Are there any other effects of citizen involvement in science in your opinion/from your experience that are not mentioned above, but that you believe were happening in the project?

Demographic data + conclusion

(with room for the interviewee to add more on the subject if they'd felt like something important had been left unsaid).

2 – Tables with illustrative quotes [translated]

Table 1: Illustrative quotes - Extended expertise in citizen science for socially robust knowledge

Extended expertise	Element	Quote
**Involving affected citizens for issue acknowledgement**	*The involvement of affected citizens*	My partner suffers a lot from it, including mental health complaints from not sleeping, a bad mood because of… the noise, the vibrations, and so on.– C05
		We live right next to the track, right. (…) A group of us, we were in the garden, and we just couldn't continue talking. And after such a long train, you kind of forget what you were we talking about again. It's just not pleasant.– C07
		Scientists often want to look at things objectively, from a distance and without emotion, while citizens can sometimes be emotional because they speak from experience. But now, as a scientist, you are directly confronted with the citizen, with society. (...) It's easy to lose that connection when you're sitting with your colleagues behind your computer all the time, writing about the world. (...). But because of the involvement of citizens, this tendency to make things too abstract is countered and the research becomes relevant to society – S03
		I think that on the one hand, by doing science or research together with citizens, you are forced to look at many issues much more practically and to think about the local impact it can have. (...) And what benefit the research has to people in certain communities. But at the same time, it's making science or research much more relevant to citizens because it clearly addresses their stories, their issues. So I think it works both ways in that sense. – S05
	Acknowledgement of the issue	Of course, it makes a big difference for our community, that we feel we can have a say, that our complaints are taken seriously . – C02
		This is something we've done together. We've discussed everything together, like 'what do you want, what do you need from us?'(...) That resulted in something we are a part of as well, something that we contributed to. That feeling of doing this together and being acknowledged, that makes so much difference compared to... like people who just sit on their throne... - C05
		Because people recognized their experiences in the final results, this research also feels as a form of acknowledgment of their problems. And that is very important. Because up till now, all they've heard is 'our methods say you don't have a problem, so stop complaining'. (...) And they get angry because they are not getting their problems acknowledged. – S02
**Knowledge exchange and**	*Community*	I think it's good for the community spirit, observing things together. And that you have a kind of gauge of what the acceptance threshold is for others – C10

**interaction between (citizen-)scientists**		Yes, I think a sense of community also develops. (...) It creates a connection for people. – S04
	Equality in collaborationg	Yes, otherwise it's more like being told from above what you should do, or what you are going to do, and having to accept it without question. – C03
		We could ask questions whenever we wanted and the explanations were clear. So, what more could we want? We even exchanged datasets. – C08
		I find it very interesting citizens also explained things to the scientists. (...) Ultimately, I think it boils down to a more balanced collaboration, a more equitable collaboration that harnesses the strengths of citizens and professional scientists.– S03
	Citizens feeling or making themselves small compared to scientists	When I see what my family member does as a scientist, no. No, I think citizen scientist is too grand a term for us. – C03
		I admire the patience of the researchers who visit here. (...) When we're at a meeting, I sometimes feel vicarious shame for my fellow-residents and how they present themselves. (...) There's always someone who wants to be kind of a smartass (...) I don't mean people are necessarily dumb, not at all. It's just... (...) Or those people who insist on speaking in dialect. (...) For God's sake, speak Dutch.' (…) Or when people ask the same question over and over again, which has already been answered, I think 'listen for once'. – C06
		Science? No, I think that's something different.- C08
**Barriers to extending the expertise**	*Skepticism from peers & time and effort*	I still have conversations with colleagues who believe that science, conventional science, is the way forward and provides the answers. It has a nice, clear framework and that's it. And some also think: "What do these citizens have to contribute? They don't really know what they're talking about." Things like that. – S01
		It also takes a lot of time and effort. And initially, you don't get funding for that. That is, of course, another problem scientists face (…) you are overburdened. You have to do many things, you have to do them all properly, and you want to do them well. – S02
	Inclusivity	The discussion often arises about whether citizen science is inclusive, whether all perspectives are heard. And that is very important. And at the same time, it is not a reason not to do it. In a lot of non-participatory scientific research, this isn't the case as well. Citizen science seems to be much more strongly scrutinized than regular science in this aspect. – S01
		With citizen sensing, it's often about technology (...)- our citizen scientists for the most part are male, on the older side, well-educated and white - S02
		I felt very involved. (...) But I don't have a lot of time available. I've noticed other people who are less busy, who take on a much more active role in the project. That's a good thing. – C06
	Balancing community involvement and scientific rigor	Because citizen scientists can be very smart and sharp about local effects, and they are increasingly involved in recognizing those in data analysis. But we also see that sometimes they make mistakes, which is very understandable if you're not trained for it. – S01

		Often it's people who live nearby, or who are affected, often they can be very critical, so to speak. People can get angry (…) and also have very sharp remarks about how we report, for example, or write things down, or the way we place sensors, or what data we use. – S03
		It's also quite challenging for scientists because you have to relinquish some control [to the citizens]. This control, as I mentioned earlier, is sometimes necessary to ensure that biases and other issues don't arise. I didn't expect this initially, but it's something I've noticed throughout the process. – S04

Table 2: Illustrative quotes - real world setting in citizen science for socially robust knowledge

Real World Setting	Element	Quote
**Measurements by a citizen science network vs establishing sound levels or calculating noise**	*Registering the noise in general*	It's better sometimes to just measure it. So you also have it on paper and can see: look, this is what we experience and that corresponds with the measurements." – C11
		Now we see how loud it is, in black on white. Before, we had to guess. (…) – C04
	Modelling not applicable in the (local) context where it is used	What happens when they do those calculations? They start considering a trajectory of a year. How much noise is produced over a year? And they take the average of that, (...) That produces a number, which supposedly indicates the noise I'll hear. But the problem is, I don't hear what is being calculated. I hear what I'm measuring, which always deviates heavily from what the model says. (…) It is not representative of what I'm experiencing. – C05
		But those calculation models just aren't correct, period. (…) If you have zero and one hundred, then you get an average noise exposure of fifty... (…) all the while you keep hearing them and everything vibrates all over the place. - C01
		I think my own measurements – I mean the meter that's hanging here - are more reliable because they really measure what's happening in that moment, instead of calculating or modeling the sounds – C06
		Well, they're all very theoretical, aren't they? In practice, it's also about the distance from your house to the railway tracks, and in what way the house itself is constructed – C10
	The models/calculations for noise are perceived as untrustworthy	[Railway organization] is kind of marking their own homework, so to say. We have a lot of doubts about them. Also because they absolutely did not want to talk about those decibels. They dismissed us, just like that. - C08
		Those calculation models can do whatever, but the sensors don't lie. – C04
**Sound measurements reflecting the real-world setting**	*Peak levels and frequency noise*	You can see those peaks regularly, which makes clear how many trains actually pass by. – C01
		Everyone has their own microphone that produces data, and you can be sure that data is accurate. (...) I can check it through an app. I can just check what… Especially last night, around four o'clock, I was awake and a huge train went by. I can check on the app, if I want to, how loud that train was – C10

		I thought: darn it, that was a long or heavy one. And then I looked and it was a confirmation of my experience. – C03
	Distinguishing train noise from other environmental noise	What I found very annoying was the remark: 'Yes, but that noise could also be a tractor, a truck combination driving on the other side of the track and they can produce high sound levels as well' (...) But now, through this project, we can see them. {*in the project we developed a method to distinguish train noise from other environmental noise sources*} – C06
		Indeed with those filters... the other traffic is removed. That was indeed something I had hoped would happen. It's nice this has been achieved. – C02
	Sound is measured on different locations	And that is a very big advantage of the method. We applied sensors on the building facade, but also in different places, and that also gave a good result. At my place, the train drives practically through the garden, but we also hung meters at people’s homes who live 20, 30, 40, 50 meters from the railway line. You see a measurable and tangible difference. - C05
		I think that our own measurements are very important and I trust those, because the sound meter is hanging both at the garage behind the house, and inside the house as well. – C06
	Sound is measured in different circumstances	Look, if there's a period when it's busy on the track or trains are moving slower, then there's much less noise. Yesterday it was busy on the track and then an iron ore train passed by, a long one, but it was driving slowly. We were all sitting outside, and we said to ourselves, ‘if they’d just go a little slower, that would improve things a lot.’ – C07
		The problem is, when we sit outside in the garden, or when we open the doors in summer and want to do something, the trains become annoying. – C08
	Adding citizen real world knowledge during analyses	Because those sensors are at their homes all the time, it's actually a kind of manned measurement. So basically, when strange things happen, citizens know that the measurement isn’t correct and they can report that, for example because there is another sound source, and then we can take that into account. Or when, at a certain moment no trains unexpectedly ran because of track work, residents immediately noticed that and told us like: hey, there were no trains last night, you can use that as a baseline measurement to see how the situation is when there are no trains running. We couldn't have seen that as quickly ourselves. So in many ways, citizens are an important part of this. – S03
**Combining sound level measurements and perception data**	*Importance of perception data*	You need to get a realistic picture of a situation, with scientific research and measurements and so on. But I think experiences are an important part as well. With those, you get a much better and accurate picture of how the situation really is. – C07
		If the residents participate, you’ll get their experiences, and in this case measurements as well. I think the experiences and the perceptions of people were just as striking and significant. The study on how people experience and perceive the noise provided much insight. – C09
		Just measuring decibels and frequency is of course very different from also noting how someone experiences something. So yes, I think it's a great combination – C11

	Learning about noise annoyance	But ultimately, to properly explain the annoyance, the question was: what annoyance do you experience? People had to report that. And eventually, you want to link that to the measurements. And it turned out that the measurements at people's homes had to be combined with the annoyance. Let me put it this way: with three sound meters in the village, we could never have explained individual annoyance – S02
		What is new in particular is that we look at the individual experiences of citizens. (...) There are studies that ask very large groups of people about their experiences over the whole year of a certain noise source. So that is much more generalized, and they link those to noise models that are also more generalized. But here we really focus on the individual situation. We place a sound meter at a person's place and ask how he or she experienced it at a particular time of day. (...) We really put the magnifying glass on something else, so to speak. – S03

Table 3: Illustrative quotes - Iterative process in citizen science for socially robust knowledge

Iterative process	Element	Quote
**Short feedback loops and mutual learning**	*Getting to the heart of the matter*	Often when I talk to residents they think: if we just measure the noise, then we have the answer and we can show how bad it is, or what the situation is. But even though ‘to measure is to know’, it’s not like we didn’t know that there is noise pollution, right? (…) And when you continue the conversation, it's not really about whether it's 80 decibels or 82 decibels. It's about the fact that it happens very often, especially at night, causing people to sleep poorly, become grumpy, and struggle to function at work. So it's not so much about measuring those decibels, even though they think it is. It's much more about how it interferes with their living environment. When you talk to people, it becomes clear it's often more about the effects of the noise on their daily lives." – S01
		And we have also discussed this with the residents:, what the measurements tell us but also what they don’t tell us. It tells us something about the sound, but it doesn't tell us how it is experienced. So, we need more than that (...) . They have shown us that something more is needed – S05
		So what often happens with official sound research is that you look at sound measurements for which there are standards. You look at daily or weekly or yearly averages (…) But what we have done now is to consider: what kind of measurement should you do to predict the actual annoyance? (...) And that simply is a very different study. – S02
	Discussions between experts steering the research / mutual learning	[Because of collaborating in the project] we’ve gotten smarter, and you’ve gotten smarter too. – C07
		In devising the measurement plan, there are a few things that, if the citizens themselves hadn't thought of it, we probably would have done it differently. For example we wouldn’t have done the indoor measurements (…) But one participant said, "I have a very quiet guest room where literally no one ever comes." And we could take good measurements there, so that was very valuable. And there was also someone who wanted a sensor in front of their house and a sensor behind their house. I wouldn't have done

		that myself, but it turned out to provide an interesting view of how the sound spreads. – S03
		The way of making the data accessible has also received feedback and has been discussed in consultation with those who want to use that dataportal. (…) So yes, you immediately have a short feedback loop, so to speak. – S04
**Iterative knowledge integration and innovation**	*Building upon / refining 'old' knowledge*	And that's why we should participate in this research, because it's not just for ourselves. I'm getting older, I hope I'll live to see what happens with the research and how it'll be used. (...) We're not alone in this world. – C03
		I hope that those who create those calculation models take this kind of research into account in the update of the calculation model. (…) Because it would get closer and closer to reality. – C05
		Well, we know about the existing research and I think the policy is also based on that. So it would be a really nice addition if you can now show: yes, but we've measured in a different way now, and these are the differences. I think that will lead to new insights.. – C11
		Okay, so we have a sound model, we have sound calculations, we have sound measurements, and we have citizen [sound and perception] measurements. And how do these relate to each other? What can you do with that? If we can start having that discussion, then I think we will make progress, as a science. – S01
		Before, I mainly worked on those sound models; those were assignments commissioned by the ministry… (…) and those calculation models have certain calculation rules that are set in law, so I had to work in a more prescribed way (…) Whereas with the citizen measurements, it's all very different because a lot is unclear and it's all still quite new. (…) So in that respect, it is a completely different approach, a more open, a more exploratory approach. – S03
	Technical innovation	What many people are interested in is: how well do these citizen science sound meters measure? (…) They are not far off from official class 1 sound meters and are very useful for mapping various things. They are really quite accurate, and cheap, and because of that, you can deploy a lot of them, so you get a better picture of the situation. (…) If we would've placed two class 1 sound meters in America, we would have had much less useful information about the area. (...) And these sound meters designs are also freely, publicly available, designed by citizens. So the design of these sound meters is citizen science as well. They are also continually improved by citizens, so they keep getting better – S03
	Complexities in integrating 'old' and 'new' knowledge	The connection between the measurements we've done now and a model seems impossible. – C02
		I recently asked the sound experts: 'How does the modeled data compare to the measured data?' But it's kind of unanswerable. Because the modeled data has so many facets that the question is to what extent the model reflects reality, or the sensor data, and we actually don't know. – S01
		Citizens say, "Why do you use models and why aren't measurements just taken everywhere?" There are reasons for that, of course (…) Sometimes things just can't be done differently because it would be too expensive, for example. – S04

Table 4: Illustrative quotes – Acceptance of citizen science knowledge

Citizen science knowledge acceptance	Element	Quote
	Utilization and advocacy	And what's valuable is, you can confront an alderman with it and say: look here. We have taken measurements. (...) these are the facts, these decibels are really high here, it's written in black and white. – C04
		The more we scientifically learn about sound, the better, actually. (...) Right now, it's all according to models and calculations and politicians are fine with that, but the people who live there are the ones who suffer. – C08
		I think it only strengthens the outcomes, right? Especially when policymakers say: 'Hey, this has been verified by the RIVM so it must be reliable.' – C11
	Tensions surrounding Policy impact	[local] Policymakers have said: these are not the numbers we are going to onsider, we will stick to the calculations. So that is a bit of a shame. – C09
		That is where I think people get frustrated with citizen science. They think, in this case too, I'm going to measure, I'll show how bad it is, and then something has to change. But nothing happens. Which is super demotivating of course, and next time they'll think: why bother, participating in science is useless to us. So, a backlash can occur. – S01
		I just can't quite assess what will be done with it afterwards. What you often see in policy now, I think, is that they want citizens to participate. But the question remains, okay, how has that had any effect on the policies? And if so, where does that become visible? – S04
		You said, 'There will be a report, you [the public] will receive it, and you can do with it what you want.(...) It's not commissioned by the ministry, so they won't receive it from us necessarily.' Our thought was: you are here for society, for the Netherlands. And if you discover something, then it's your responsibility to pass it on– C01
